# Mutagenesis Objective Search and Selection Tool (MOSST): an algorithm to predict structure-function related mutations in proteins

**DOI:** 10.1186/1471-2105-12-122

**Published:** 2011-04-27

**Authors:** Alvaro Olivera-Nappa, Barbara A Andrews, Juan A Asenjo

**Affiliations:** 1 Centre for Biochemical Engineering and Biotechnology, Institute for Cell Dynamics and Biotechnology: a Centre for Systems Biology, University of Chile, Beauchef 850, Santiago, Chile

## Abstract

**Background:**

Functionally relevant artificial or natural mutations are difficult to assess or predict if no structure-function information is available for a protein. This is especially important to correctly identify functionally significant non-synonymous single nucleotide polymorphisms (nsSNPs) or to design a site-directed mutagenesis strategy for a target protein. A new and powerful methodology is proposed to guide these two decision strategies, based only on conservation rules of physicochemical properties of amino acids extracted from a multiple alignment of a protein family where the target protein belongs, with no need of explicit structure-function relationships.

**Results:**

A statistical analysis is performed over each amino acid position in the multiple protein alignment, based on different amino acid physical or chemical characteristics, including hydrophobicity, side-chain volume, charge and protein conformational parameters. The variances of each of these properties at each position are combined to obtain a global statistical indicator of the conservation degree of each property. Different types of physicochemical conservation are defined to characterize relevant and irrelevant positions. The differences between statistical variances are taken together as the basis of hypothesis tests at each position to search for functionally significant mutable sites and to identify specific mutagenesis targets. The outcome is used to statistically predict physicochemical consensus sequences based on different properties and to calculate the amino acid propensities at each position in a given protein. Hence, amino acid positions are identified that are putatively responsible for function, specificity, stability or binding interactions in a family of proteins. Once these key functional positions are identified, position-specific statistical distributions are applied to divide the 20 common protein amino acids in each position of the protein's primary sequence into a group of functionally non-disruptive amino acids and a second group of functionally deleterious amino acids.

**Conclusions:**

With this approach, not only conserved amino acid positions in a protein family can be labeled as functionally relevant, but also non-conserved amino acid positions can be identified to have a physicochemically meaningful functional effect. These results become a discriminative tool in the selection and elaboration of rational mutagenesis strategies for the protein. They can also be used to predict if a given nsSNP, identified, for instance, in a genomic-scale analysis, can have a functional implication for a particular protein and which nsSNPs are most likely to be functionally silent for a protein. This analytical tool could be used to rapidly and automatically discard any irrelevant nsSNP and guide the research focus toward functionally significant mutations. Based on preliminary results and applications, this technique shows promising performance as a valuable bioinformatics tool to aid in the development of new protein variants and in the understanding of function-structure relationships in proteins.

## Background

Site-directed mutagenesis is a tool used in rational protein design strategies to modify the structure or function of a protein to adapt it to particular performance requirements. Moreover, mutagenesis is a fundamental tool to study the relationship between protein structure and function, making possible the substitution of one amino acid by another, thus isolating the contribution of the original amino acid or the newly introduced amino acid to the structure and function of the protein as a whole [1,2].

However, site-directed mutagenesis-based rational protein design strategies present a widely recognized drawback. In order to introduce changes that could confer a desired function or characteristic to a protein, it is necessary to know, or at least to assume, something about the protein structure-function relationship. In other words, it is necessary to know, for each amino acid of the protein or at least for a select group of them, what is their particular contribution to the structure and function of the protein as a whole.

Even for a small protein, and assuming that only a subset of amino acids contribute to features really determinant for its relevant function, the number of amino acids to be considered is very large. For a medium-sized protein and with no additional information regarding the possible structure-function relationship, an exhaustive search is practically impossible [3].

It is commonly known that there are certain amino acids in a protein that are necessary and fundamental for its activity, function or structure, and there are other amino acids that are readily replaceable by amino acids sharing a common characteristic, without affecting the main features of the protein. Therefore, certain amino acidic positions must conserve some unique properties, which are communicated to the entire molecule, for a given protein or protein family. When analyzing a protein family, many functionally important residues of proteins can be identified because they have been conserved during evolution. However, residues that vary can also be critically important if their variation is responsible for diversity of protein function and improved phenotypes. This adds an entirely new complexity level to the analysis [4,5].

The same type of functional variation can be observed in nature, represented by single nucleotide polymorphisms (SNPs) in the coding region of a given gene. SNPs can be synonymous, often called silent mutations, or can substitute a particular amino acid for another in a protein primary sequence, which is referred as to a non-synonymous SNP (nsSNP). Prediction of the occurrence of nsSNPs in a gene could be easily done by comparing nucleotide sequences and detecting or predicting nucleotide changes that occur with low-probability incidence. However, predicting which of these mutations will have an observable effect on protein function is a much more difficult task. This is complicated by the fact that there is often no structure-function knowledge available about the protein. This variation in protein function can be subtle or lead to major phenotypic changes in living organisms. For instance, variations in the DNA sequences of genes can affect how humans develop diseases and respond to pathogens, chemicals, drugs, vaccines, and other agents.

When no structure-function information is available, it is relevant to determine these features only from the protein's amino acid sequence [5]. A number of bioinformatic algorithms have been devised previously to extract this type of information from the primary sequence of a protein [4-8]. Some of these methods have been successfully used for the prediction of altered protein phenotypes caused by nsSNPs in protein genes [7-9]. Other methods have been described to derive this information from 3D structural data or molecular dynamics results [10]. A different approach that incorporates machine-learning techniques has been used to study the results of directed evolution experiments in order to explore proteins and to derive hidden structural rules [11].

For this large-scale analysis, we propose an alternative, taking into account the contribution of each amino acid to the general structure of the protein through their characteristic physicochemical properties, such as hydrophobicity, side-chain volume and charge. Each amino acid contributes with its own physicochemical characteristics to the entire protein, which adds to the characteristics of the other amino acids, thus determining the relevant features of the protein, both global and site-specific [2].

In this study, we have developed a general algorithm, named Mutagenesis Objective Search and Selection Tool (MOSST), which analyzes the target protein as part of a multiple alignment to determine which are the positions that could be mutated with or without altering the common characteristics of the protein family, and gives mutagenesis estimations related to the possibility of whether a given amino acid change would have deleterious effects on the protein. A variant of the same method can be used to detect phenotypically relevant nsSNPs in a gene family and separate them from amino acid substitutions that do not have functional implications.

## Results and Discussion

### Algorithm

**Figure 1:**
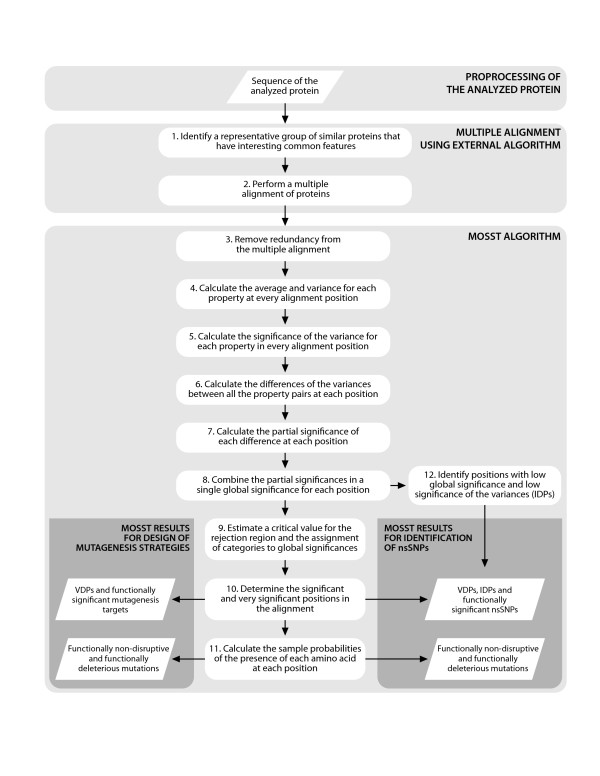
**Flow diagram of the whole MOSST algorithm**. The algorithm can be alternatively used for the development of rational protein design strategies or for the identification of functionally significant nsSNPs.

As a summary of this work, the general algorithmic procedure is presented in Figure 1. The left end set of the global algorithm corresponds to the information delivered to select and design site-directed mutagenesis strategies, while the right end set represents the key information used to identify functionally significant nsSNPs using the tools and statistical procedures proposed in this paper. Both analytic procedures share the same initial steps, but have subtle differences that are exploited to get the most appropriate results for each application.

#### Background of the algorithm

A general consensus in protein science states that some amino acid physicochemical properties are conserved at particular positions in a given protein family and some others are not. This explains the possible variation that can be found in any protein family to achieve the same function and it also shows that, despite the possibility of variation, there are particular characteristics that must be retained in order for the protein to conserve its own distinctive features.

Each macroscopic property of a protein is controlled by different amino acids in its sequence with a higher or lower degree of influence. Hence, both within the framework of site-directed mutagenesis experiments and in the detection and prediction of phenotypic effects of nsSNPs, it is interesting to identify amino acid residues that are relevant for a protein's function, specificity, stability or binding interactions.

The proposed algorithm has been designed to analyze the general conservation of each relevant physicochemical characteristic at each amino acid position in a protein family (e.g. hydrophobicity, charge, volume and shape). In this context, a protein family is predefined by selecting relevant proteins according to arbitrary criteria (e.g. a certain function in the case of an enzyme family, or the conservation of a certain structure in the case of a conserved protein fold). A protein family can also be subdivided into subfamilies, in order to study the contribution of particular amino acids to differences between subfamilies.

#### Preprocessing of the target sequence

To compare the proteins belonging to a family, a multiple alignment of the amino acid sequences is carried out. The most used algorithm for these multiple alignments is ClustalW, developed by Thompson et al. [12] In a multiple alignment, the algorithm tends to retain and conserve the amino acids in each position, using a weight matrix (for example, Blosum matrices [13] and the Gonnet matrix [14]). An optimal alignment is obtained using these general matrices, which guarantee that the properties as a whole are maximally conserved at each amino acid position of the alignment.

The preprocessing of the target protein sequence is represented by steps 1 and 2 in Figure 1. The MOSST algorithm subsequently analyzes this multiple alignment to determine and quantify the statistical significance of the conservation of different physicochemical properties in particular positions of the protein family alignment. To perform this analysis, the conservation of each property is independently studied and patterns are identified by comparing the conservation of different properties at each amino acid position. Typical conservation cases are defined based on these conservation patterns, which are then used to statistically classify the amino acids as relevant or irrelevant with respect to the conservation of any physicochemical property in the protein and the predicted amino acid mutability in the protein family. Then the statistical parameters (probabilities) determined for the conservation of each amino acid position are used as predictors to classify possible mutations in the protein family as functionally impairing or functionally silent. Hence, these results can then be interpreted and sorted to design mutagenesis strategies or to identify nsSNPs.

#### Control of the quality of the multiple alignment

The proposed method allows identification of functionally relevant positions in a particular protein when the protein is placed within a comparative reference group in a multiple alignment. The classification procedure gives unique results for each particular alignment, but the statistical result for each position will strongly depend on the quality of the alignment and nothing prevents *a priori *this significance level varying between one alignment and another.

This variation must be controlled so that the comparison gives coherent and reproducible results when confronted to minor changes in the comparison alignment. The proposed control of this variation, represented by step 3 in Figure 1, is done in two ways:

##### Alignment quality control

The quality of a multiple alignment specifically depends on the substitution matrices used and the negative weight contributions of gap creation and extension. In the test performed to check the proposed algorithm, the alignments obtained using default ClustalW parameters gave satisfactory results.

##### Control of the representativity and the redundancy of the alignment

In the proposed analysis algorithm, the group of proteins in the alignment is supposed to be representative of a wider protein group. If the protein group does not include representatives of some subgroup that has some special feature, then such feature evidently cannot be included in the analysis. This sub-representation is impossible to detect by purely mathematical methods, but can be solved by judiciously choosing the proteins to be represented and analyzed, which is a task that has to be done by a suitable expert. On the other hand, if a subgroup of proteins is over-represented in the analyzed group, then the protein sample will be biased towards the characteristics of the most represented subgroup, ignoring the characteristics of the other subgroups. The over-representation of a subgroup in the sample is readily detectable, as it is possible to assess the percentage of similarity between all the proteins in a group. If a particular group shows a high similarity degree, i.e. high redundancy, then it is over-represented in the sample, and the number of proteins in that group could be reduced by eliminating the most similar proteins.

Once a representative and non-redundant group of proteins is found, MOSST can be used to detect functionally relevant positions.

#### Statistical quantification of the conservation of properties

Amino acids have physical and chemical properties that can be directly or indirectly measured, e.g. hydrophobicity, polarity, charge, molecular shape, conformational propensity and others. A numerical value is typically assigned for the measured property to each amino acid, whether free or associated to others in a polypeptide chain. As side chains are different for each of the 20 naturally occurring amino acids, these numerical values are different for each amino acid and provide a quantitative ordering between them with respect to the measured property. Hence, these property values form a scale that represents a numerical scoring for each amino acid. Many of these amino acid properties have been compiled in multiple non-independent "amino acid scales" [15-50].

In this way, every amino acid is assigned a numerical score that quantifies a particular physicochemical property **Ω **(for nomenclature, see Table 1). Hence, any group of ***n ***amino acids will have ***n ***associated scores, ***X*_Ω,1_...*X*_Ω,*n*_**. From these scores, an arithmetical average ***μ*_Ω _**and a sample variance estimator ***σ*_Ω _**can be calculated (Figure 1, step 4) as:(1)(2)

**Table 1:** Nomenclature and abbreviations

Variable name	Description
	Posterior cumulative distribution function for the values of the property **Ω **at position ***i***, given the previously known group of ***n ***amino acids present in the multiple alignment at such position
**C*DF*_(Ω1,Ω2),*n*_**	Cumulative distribution function of the absolute difference between the variances of any two properties **Ω1 **and **Ω2 **for a random combination of ***n ***amino acids
***CDF*_Ω,*n*_**	Cumulative distribution function for a random combination of ***n ***amino acids and a given property **Ω**
***H_0_***	Null hypothesis in the hypothesis test for differences
***H_1_***	Alternative hypothesis in the hypothesis test for differences
***i***	Position number in the multiple alignment
**IDP**	Invariable determinant position
***j ***or ***k***	Amino acid numbering sub-index
***L***	Total number of physicochemical properties considered in the MOSST analysis
***M***	Number of possible pairwise combinations (subsets having two elements) of properties
***n***	Number of amino acids in a multiple alignment position (without taking gaps into account)
***N***	Total number of positions in the multiple alignment
***N(μ,σ)***	Normal distribution function with unknown parameters ***μ***(mean) and ***σ ***(variance)
***n_i_***	Number of amino acids in position ***i ***of the multiple alignment (without taking gaps into account)
**nsSNP**	Non-synonymous single nucleotide polymorphism
***p'*_*i*,(Ω1,Ω2),*n*_**	**= 1 - *p*_*i*,(Ω1,Ω2),*n*_** Level of significance of the null hypothesis ***H_0 _***for the difference of the variances of the properties **Ω1 **and **Ω2 **at position ***i ***of a multiple alignment containing ***n ***amino acids
***p'*_*i*,(Ωr,Ωs),*n*_**	**= 1 - *p*_*i*,(Ωr,Ωs),*n*_** Level of significance of the null hypothesis ***H_0 _***for the difference of the variances of the properties **Ωr **and **Ωs **at position ***i ***of a multiple alignment containing ***n ***amino acids
***p'_m_***	Value of the ***m***^th ^level of significance, obtained from the set of ***M ***levels of significance of the differences between variances for all the possible pairwise combinations of properties, sorted in ascending order, for a given position ***i ***of a multiple alignment
***p*_(Ω1,Ω2)_**	Probability of getting a certain value or less for the absolute difference ***Δσ*_(Ω1,Ω2) _**between the variances of any two properties **Ω1 **and **Ω2**
	Posterior probability distribution function for the values of the property **Ω **at position ***i***, given the previously known group of ***n ***amino acids present in the multiple alignment at such position
***P_i_***	Global probability of the null hypothesis being true considering all the properties together at position ***i**, i.e*. probability of all the variances being equal at position ***i ***of a multiple alignment
***p*_*i*,(Ω1,Ω2),*n*_**	Probability of getting a certain value or less for the absolute difference ***Δσ*_(Ω1,Ω2) _**between the variances of any two properties **Ω1 **and **Ω2 **at position ***i ***of a multiple alignment containing ***n ***amino acids
***p*_*i*,(Ωr,Ωs),*n*_**	Probability of getting a certain value or less for the absolute difference ***Δσ*_(Ωr,Ωs) _**between the variances of any two properties **Ωr **and **Ωs **at position ***i ***of a multiple alignment containing ***n ***amino acids
***p*_*i*,Ω,*n*_**	Cumulative probability of getting a certain value or less for the variance (***σ*_*i*,Ω,*n*_**) for a given property **Ω **in position ***i ***of a multiple alignment containing ***n ***amino acids
***PMF*_Ω,*n*_**	Discrete probability mass function for a random combination of ***n ***amino acids and any given property **Ω**
***p*_Ω_**	Cumulative probability of getting a certain value or less for the variance for a given property **Ω**
***Q_j,i_***	Global probability for the occurrence of the amino acid ***j ***considering all properties together at position ***i ***in the multiple alignment, *i.e. *probability of the amino acid ***j ***satisfying all the physicochemical requirements at position ***i***
***q*_*j,i*_**,_Ω,***n***+1 _or	Probability of the amino acid ***j ***(any of the 20 natural amino acids) to be present at position ***i ***(containing ***n ***amino acids in the multiple alignment analyzed) in a new protein not included in the multiple alignment, according to property **Ω **or **Ω*_r_***
***r ***or ***s***	Property numbering sub-index
**VDP**	Variable determinant position
**VIP**	Variable irrelevant position
***x***	Unknown quantity or variable
***X***_***i***,Ω,***j***_ or	Measure, value or score of the physicochemical property **Ω **or **Ω*_r _***for the amino acid ***j ***at position ***i ***of a multiple alignment
***X*_Ω,*j*_**	Measure, value or score of the physicochemical property **Ω **for the amino acid ***j***
***Δσ*_(Ω1,Ω2)_**	Absolute difference between the variances of any two properties **Ω1 **and **Ω2**
***Δσ*_*i*,(Ω1,Ω2),*n*_**	Absolute difference between the variances of any two properties **Ω1 **and **Ω2 **at position ***i ***of a multiple alignment containing ***n ***amino acids
***Δσ*_*i*,(Ωr,Ωs),*n*_**	Absolute difference between the variances of any two properties **Ωr **and **Ωs **at position ***i ***of a multiple alignment containing ***n ***amino acids
***μ***	Mean (of a normal distribution)
***μ*_*i*,Ω,*n*_**	Arithmetical average of the physicochemical property **Ω **in position ***i ***of the multiple alignment containing a number of ***n ***amino acids
***μ*_Ω_**	Arithmetical average of the physicochemical property **Ω**
***σ***	Variance (of a normal distribution)
***σ*_*i*,Ω,*n*_**	Sample variance estimator (standard deviation) of the physicochemical property **Ω **in position ***i ***of the multiple alignment containing a number of ***n ***amino acids
***σ*_Ω_**	Sample variance estimator (standard deviation) of the physicochemical property **Ω**
***τ*_*i*,Ω,*n*+1_**	Test statistic for the (***n*+1**)^th ^amino acid at position ***i ***of a multiple alignment containing ***n ***amino acids
**Ω**	Generic physicochemical property
**Ω1**, **Ω2**, **Ω3**	Physicochemical properties 1, 2 and 3
**Ωr**, **Ωs **or **Ω*_r_***, **Ω*_s_***	Physicochemical properties ***r ***and ***s***

**Figure 2:**
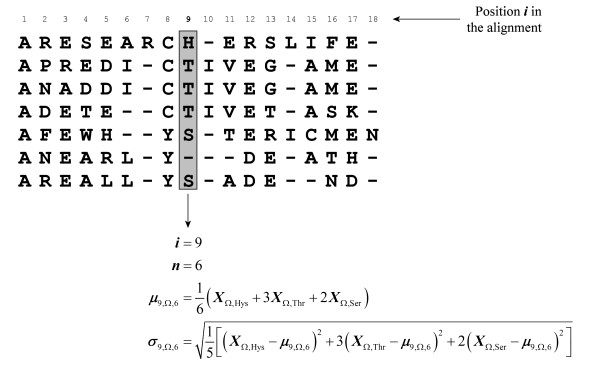
**Example multiple alignment of seven protein amino acid sequences**. Each example protein has 16, 15, 15, 14, 15, 12 and 13 amino acids, respectively. The multiple alignment has a length of 18 positions, which means that every sequence has at least 2 gaps. The calculation of the mean and variance of the property **Ω **is shown in detail for position 9 of the alignment (***i ***= 9), with 6 amino acids and one gap (***n ***= 6: 1 histidine, 3 threonines and 2 serines).

A multiple alignment of protein amino acid sequences, such as that obtained by using ClustalW, comprises a given number of ***N ***positions. Each position ***i ***contains a group of ***n_i _***optimally aligned amino acids, each from a different protein (Figure 2). As the multiple alignment at position ***i ***can include gaps, ***n_i _***is not always equal to the number of proteins in the alignment. For example, in the ninth position (***i *= 9**) of the alignment in Figure 2, ***n*_9 _= 6**. Hence, for any given amino acid property **Ω**, an average ***μ*_*i*,Ω,*n *_**and a variance ***σ*_*i*,Ω,*n *_**can be calculated for each position ***i ***in a multiple alignment with ***n ***amino acids, using Eq.1 and Eq.2, respectively.

The variance ***σ*_*i*,Ω,*n*_**, as its name suggests, quantifies the variability of the property **Ω **in position ***i***. If ***σ*_*i*,Ω,*n *_**is small, a large variation in the property **Ω **is not allowed in position ***i***, and *vice versa*. Therefore, ***σ*_*i*,Ω,*n *_**can be used to quantify the conservation of the property **Ω **at that position. However, ***σ*_*i*,Ω,*n *_**depends on the scale used to measure **Ω **and on the number ***n ***of amino acids compared. Hence, it cannot be used to compare the conservation of different properties or different quantification scales, or to compare the conservation between positions with different numbers of amino acids. A different scale-free and position-free standard parameter has to be calculated to quantify and compare the conservation of properties in multiple alignments.

In the general case, there are 20 different amino acids and therefore for a given number of ***n ***amino acids there are **20*^n ^***different random amino acid combinations. For any given property, it is possible to calculate all the **20*^n ^***possible combinations of ***n ***amino acids and their associated averages and variances. If amino acid combinations are random, any particular combination will have a probability of occurrence equal to **1/20*^n^***. Therefore, both the average and variance associated to each combination will have a probability of occurrence equal to **1/20*^n^***. Particularly, for a random combination of ***n ***amino acids and any given amino acid property **Ω**, a discrete probability mass function (***PMF*_Ω,*n*_**) for the variance can be constructed. The function ***PMF*_Ω,*n*_(*σ *= *x*) **gives the probability that the variance ***σ ***of the property **Ω **calculated for a group of ***n ***amino acids is exactly equal to ***x ***[51].

**Figure 3:**
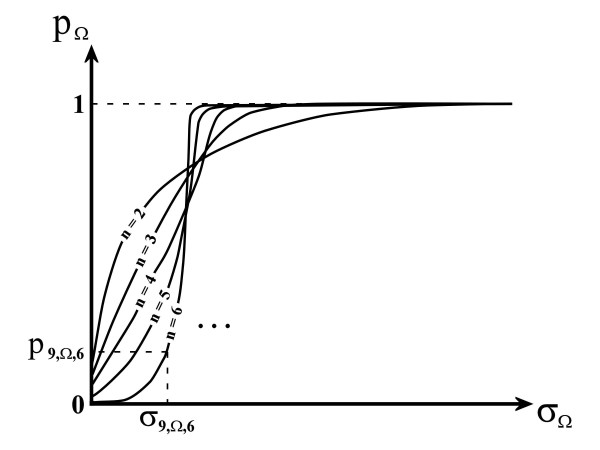
**Typical plots of the cumulative distribution functions (CDF) of sample variances**. In these plots, ***σ*_Ω _**is a calculated sample variance for any combination of ***n ***amino acids and ***p*_Ω _**is the associated probability of obtaining such sample variance value for the property **Ω **just randomly choosing ***n ***amino acids, and CDF profiles vary depending on the number of amino acids selected. Continuing with the example of Figure 2, the sample variance ***σ*_9,Ω,6 _**has an associated probability ***p*_9,Ω,6 _**that can be found using the corresponding ***CDF*_Ω,*n*_**.

From the ***PMF*_Ω,*n *_**for the variance of the property **Ω**, a cumulative distribution function (***CDF*_Ω,*n*_**) can be obtained [51]. As shown in Figure 3, if the variance ***σ*_Ω _= *x***, then ***CDF*_Ω,*n *_(*σ*_Ω _= *x*) = *p*_Ω _**is the probability of the variance of a random combination of ***n ***amino acids being between 0 and ***x***. Thus, if ***p*_*i*,Ω,*n *_**in a given position ***i ***in a multiple alignment is small, then the probability of said variance having a value equal or less than ***x ***is very small in comparison with the probabilities of all the possible variances in a random combination of ***n ***amino acids, and *vice versa*. For example, a value of ***p*_*i*,Ω,*n *_= 0.05 **means that the obtained variance ***σ*_*i*,Ω,*n *_**(or a lower value) only occurs in a proportion of 1 to 20 (1/20 = 0.05) in random combinations of ***n ***amino acids.

The significance of variances for every position ***i ***in the target multiple protein alignment are calculated in step 5 of the algorithm (Figure 1). Notably, if ***p*_*i*,Ω,*n *_**in a given position ***i ***in a multiple alignment is small, the variance ***σ*_*i*,Ω,*n *_**of the amino acid property under analysis in that position is simultaneously small and relatively uncommon, *i.e. *the amino acid selection in that position is not random. Hence, a small ***p*_*i*,Ω,*n *_**implies that the property value must be relatively invariable (*i.e. *conserved) at position ***i***, and *vice versa*. The closer to 0 is the value of ***p*_*i*,Ω,*n*_**, the more significant this variance is and the less random the amino acid group is at position ***i***.

The probability ***p*_*i*,Ω,*n *_**is an indicator of the degree of conservation of any property **Ω **at each position ***i ***in a multiple alignment, contrary to the use of the variance alone. The advantages of this conservation measure are, firstly, its independence with regard to the number ***n ***of amino acids in the comparison (including the presence of gaps) and, secondly, the possibility of performing scale-free and position-free comparisons between conservations at different positions and using different scales or properties. Interestingly, the probability value ***p*_*i*,Ω,*n *_**could also be used as a significance value in hypothesis tests for the obtained variance ***σ*_*i*,Ω,*n*_**.

#### Determination of mutationally relevant positions

**Figure 4:**
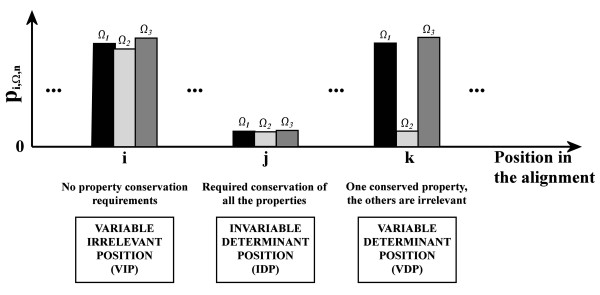
**Scheme depicting the three possible conservation cases described in the text**. For each different position (***i**, **j**, **k***) of a multiple alignment, the significance levels ***p*_9,Ω,6 _**corresponding to three amino acid properties of the example in Figure 2 (***Ω_1_**, **Ω_2_**, **Ω_3_***) are plotted, to determine the differential conservation of the properties.

Ideally, each property **Ω **is unique and mutually independent, and its contribution to the global structure and function of a protein is relatively important. For an amino acid position ***i ***in a multiple alignment, and given a cumulative probability ***p*_*i*,Ω,*n *_**for each amino acid property, we define three possible cases into which the relationships between the different probabilities ***p*_*i*,Ω,*n *_**for each amino acid property at a position ***i ***could be classified *a priori*. In Figure 4, these three possible cases are shown with reference to three different amino acid scales. These three cases, which are defined in the following paragraphs, can be used as a basis to determine good mutagenesis objectives.

##### Invariable determinant position (IDP)

The first case is defined by obtaining a high significance level, *i.e. *a very low ***p*_*i*,Ω,*n *_**value, for every amino acid property in a position ***i***. This implies a relevant conservation of all the properties at that position. These positions are usually identified in multiple alignments as those positions that stringently accept only one or sometimes two different specific amino acids and are thus readily identifiable and visible using simple methods. The change of one amino acid for another in the analyzed position would probably imply a drastic change in the value of one or more properties and therefore alter the conserved necessity for invariability at that position, causing a partial or total loss of some of the characteristic properties of the protein family. If it is desired to conserve such properties, e.g. conservation of catalytic function or interaction sites, as would be the general case in conservative site-directed mutagenesis studies, then these positions should not be considered as possible mutagenesis objectives. A mutation in this type of position is usually deleterious for the protein function in nsSNPs.

##### Variable irrelevant position (VIP)

The second typical case is when all the probabilities ***p*_*i*,Ω,*n *_**associated to variances for all the amino acid properties at position ***i ***are very high. Here, the significance of conservation of those properties at that position is very low, *i.e. *conservation is irrelevant. The replacement of one amino acid by another in any of these positions should not be determinant for the protein family, as the conservation of any particular characteristic is not required. In other words, the amino acids present at these positions do not contribute fundamentally to any relevant characteristic of the protein family. Therefore, it is expected that mutagenesis at a variable irrelevant position should not have a large effect on the characteristics of a protein family. A nsSNP with these characteristics is probably silent and does not affect protein function.

##### Variable determinant position (VDP)

The third case corresponds to the case where, at the analyzed position ***i***, there is a high conservation, i.e. a high ***p*_*i*,Ω,*n *_**significance, in one property and a very low significance or conservation in the remaining properties. Then, for this position there is a very high tendency to conserve one of the independent amino acid properties, which are necessarily privileged, and a simultaneous tendency to variability in the other groups of rather irrelevant properties. Thus, this position is variable in the sense of admittance of global variability of some characteristics, but nevertheless is determinant for possession of a conserved group of characteristics, which have to be present to communicate common characteristics to the protein family. This makes this kind of position a main target to be mutated. A nsSNP in this type of position can affect protein function in a rather unpredictable but probably very determinant way.

A conservative mutagenesis strategy that aims to preserve the main functional or interactional properties that characterize the protein family and to selectively alter secondary traits should be focused on mutating only VDPs, while a non-conservative mutagenesis strategy could also include IDPs. If the aim is to identify nsSNPs, mutations that could alter protein function are most probably located at IDPs and VDPs. Whichever the task and the expected result would be, it is evidently useful to identify VDPs, given their allowed amino acid variability and the simultaneous relative importance of conservation of one property in such position in the entire protein family. However, VDPs are much more difficult to find than IDPs, which makes VDPs prime "hidden" functionally relevant positions that cannot be readily identified by other existing automatic methods. The following sections will describe the proposed method to identify these positions.

#### Identification of Variable Determinant Positions

We described previously that the determination of the significance values ***p*_*i*,Ω,*n *_**for each position ***i ***in a multiple alignment is based on the CDFs of the variances of random combinations of ***n ***amino acids. After CDFs are calculated for the variance of each amino acid property, the significance ***p*_*i*,Ω,*n *_**associated to each property can be calculated at each position.

With these ***p*_*i*,Ω,*n *_**values we can identify in an alignment the VDPs described previously as hidden functionally determinant positions where one of the properties is conserved, while others are not. To identify such positions (Figure 1, step 10), it is necessary to determine whether significant differences exist between the variances of the different amino acid properties at a given position ***i***. In fact, differences between the variance of one property and the variances of every other could be used as indicators of the presence of conservation of a single property in position ***i***. However, only significant (not random) differences will indicate that position ***i ***is indeed a VDP. Hence, in the following section, we develop a method that uses hypothesis testing to identify VDPs by quantifying the magnitude of the significance of such differences and assigning a probability to classify position ***i ***as a VDP.

#### Hypothesis test for differences

**Figure 5:**
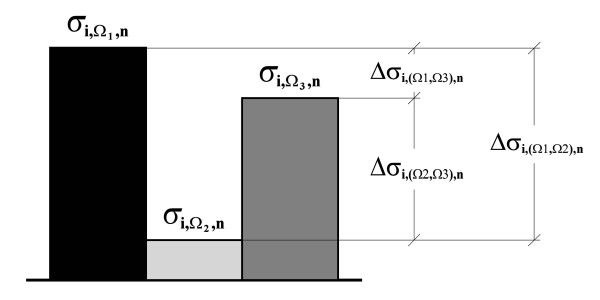
**Scheme depicting the calculation of differences *Δσ*_*i*,(Ωr,Ωs),*n *_between pairs of sample variances for a VDP**. Large paired differences exist maximally only when one property is strictly conserved while the others are not. This is exploited to combine evidence by integrating individual significances from different pairs of properties.

**Figure 6:**
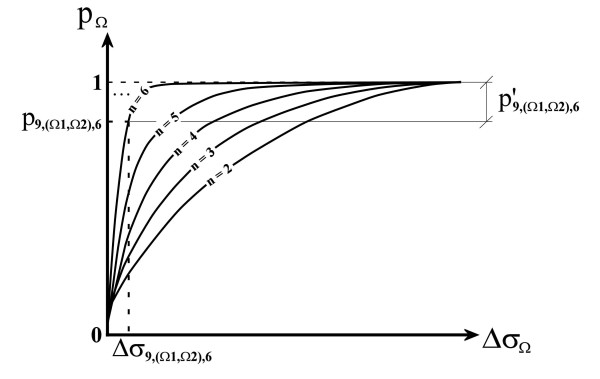
**Typical plots of the cumulative distribution functions (CDF) of the differences of sample variances**. In these plots, **Δ*σ*_Ω _**is a calculated difference between sample variances for any combination of ***n ***amino acids and ***p*_Ω _**is the associated probability of obtaining such difference of sample variances for the property **Ω **just randomly choosing ***n ***amino acids, and the CDF profiles vary depending on the number of amino acids selected. Continuing with the example of Figure 2, a calculated difference **Δ*σ*_9,(Ω1,Ω2),6 _**of the sample variances between the properties **Ω_1 _**and **Ω_2 _**at position ***i ***= 9 and ***n ***= 6, has an associated probability ***p*_9,(Ω1,Ω2),6 _**that can be found using the corresponding ***CDF*_(Ω1,Ω2),*n*_**. Therefore, the significance level of the null hypothesis ***H_0 _***is ***p'*_*i*,(Ω1,Ω2),*n *_**= **1 - *p*_*i*,(Ω1,Ω2),*n*_**.

In the same way that probabilities ***p*_Ω _**can be calculated and cumulative distribution functions can be constructed for the variance of any given property **Ω**, a probability ***p*_(Ω1,Ω2) _**for all the possible differences ***Δσ*_(Ω1,Ω2) _**between the variances of any two properties **Ω1 **and **Ω2 **can be found (Figure 5). The probability value ***p*_(Ω1,Ω2) _**is equivalent to the probability of the absolute difference between variances being ***Δσ*_(Ω1,Ω2) _**or less. From these differences and their associated probabilities, a corresponding ***CDF*_(Ω1,Ω2),*n *_**can be calculated for a group of ***n ***amino acids, considering all the possible random combinations of variances and their differences (Figure 6).

Therefore, for every position ***i ***with ***n ***amino acids in a multiple alignment, a hypothesis test can be devised to test whether a given difference between the variances of two given properties **Ω1 **and **Ω2 **is not random, *i.e. ****Δσ*_*i*,(Ω1,Ω2),*n *_**is significant. The null and alternative hypotheses for this test are, respectively:

***H_0_***: The variances of both amino acid properties are equal (*i.e. *their difference ***Δσ*_*i*,(Ω1,Ω2),*n *_**is random).

***H_1_***: The variances of both amino acid properties are different (*i.e. *their difference ***Δσ*_*i*,(Ω1,Ω2),*n *_**is not random).

Thus, ***CDF*_(Ω1,Ω2),*n *_**is the distribution function of the test statistic ***Δσ*_*i*,(Ω1,Ω2),*n *_**under the null hypothesis, *i.e. *random differences between the variances. Hence, ***CDF*_(Ω1,Ω2),*n *_(*Δσ*_*i*,(Ω1,Ω2),*n *_= *x*) = *p*_*i*,(Ω1,Ω2),*n *_**is the probability of the difference between the variances of two properties **Ω1 **and **Ω2 **in a random combination of ***n ***amino acids being less than ***x ***(see the example in Figure 6). Therefore, ***p'*_*i*,(Ω1,Ω2),*n *_**= **1 - *p*_*i*,(Ω1,Ω2),*n *_**is the probability (or level of significance) of the null hypothesis for the properties **Ω1 **and **Ω2 **(Figure 6). For very low values, under certain arbitrary limits, the null hypothesis is not significant and could be rejected in favor of the alternative hypothesis.

The partial significance of the differences between each pair of properties is calculated in step 7 of the algorithm. However, as there are many different properties **Ω**, for each position ***i ***in a multiple alignment, each pair of properties will have a corresponding significance value for the difference between their variances. None of these differences or their related significance levels could be seen mathematically as the significance of the position by itself. The significance of the position must integrate all the individual significances in a global significance value. If the null hypothesis is significantly rejected for one single property when compared with all the other properties, position ***i ***can most likely be identified as a VDP. In particular, to identify a VDP the global significance must be maximal when all differences except one are significant.

#### Integration of the individual significances

For a given position ***i ***in the alignment comprising a group of ***n ***amino acids, every possible pair of properties **Ωr **and **Ωs **will have a difference ***Δσ*_*i*,(Ωr,Ωs),*n *_**between their respective variances, and a significance level ***p'*_*i*,(Ωr,Ωs),*n *_**associated to this difference. Each comparison between any pair of properties gives information to prove or disprove that the variances of the properties are different. Therefore, every ***p'*_*i*,(Ωr,Ωs),*n *_**can be considered as the significance level of a single experiment or sampling test to reject the null hypothesis at position ***i***. However, the level of significance of the global null hypothesis must take into account the level of significance of all the differences between every pair of property variances. Therefore, none of the ***p'*_*i*,(Ωr,Ωs),*n *_**alone is able to represent the significance of the position ***i ***on its own.

Statistically, the significance levels obtained from different experiments to demonstrate the same hypothesis could be combined in order to get a composite level of significance, as if a single experiment would be done with the combined evidence of all those partial experiments. This combination could be done using different procedures according to the features of the particular cases (e.g. the Fisher procedure [52,53], procedures based on the Bonferroni inequality [54,55] and the improved Simes procedure [55,56]). In our case, the differences of variances between each possible pair of properties are not independent, since the difference between the last pair of properties is a linear function of the others, so the Fisher procedure cannot be used. From the other two methods, the Simes procedure gives the best results, as its rejection region contains the rejection region of the Bonferroni methods and it is always more powerful than those, specially in the case of highly dependent test statistics [56], which is suspected to be our case.

We propose the use of the Simes method to combine the significance values of the differences of variances between all the properties, in order to obtain a global significance value for the differences at position ***i ***(Figure 1, step 8). The Simes method can be applied in a simple way: if ***p'*_*m *_= *p'*_*1*_**, ***p'*_*2*_**,... ***p'*_*M *_**is the value of the level of significance of each difference of variances between all the pairwise combinations of properties, in ascending order, the value of the global significance that considers all the individual partial significances for position ***i ***is:(3)

The global probability ***P_i _***represents the probability of the null hypothesis being true, given the values obtained for the differences of variances between different properties. In our case, ***P_i _***is the probability of all the variances being equal. The lower this probability, the higher the accuracy with which the null hypothesis could be rejected, i.e. it is more probable that the variances of the different properties at position ***i ***in the analyzed multiple alignment could be different. Hence, low ***P_i _***values could be used to directly identify VDPs in a multiple alignment (step 10, Figure 1).

#### Determination of the rejection regions for the null hypothesis

Statistical practice classifies the levels of significance of a hypothesis test in different categorical levels, usually "not significant", "significant" and sometimes "very significant". This categorization is performed using a criterium that imposes an arbitrary limit between one significance level and another. Usually, a value of 0.05 is the limit between "non significant" and "significant" levels. However, multiple experimental implementations of the proposed algorithm indicate that this limit is not stringent enough to correctly classify VDPs apart from IDPs and VIPs. Preliminary evidence shows that positions in a multiple alignment could be roughly classified into one of the following categories according to their significance levels: not significant (***P_i _***> 0.01), significant (0.0005 <***P_i _***≤ 0.01) or very significant (***P_i _***≤ 0.0005).

With this classification, critical values for the rejection region of the null hypothesis are determined (Figure 1, step 9) and VDPs in the target protein are those positions identified as significant or very significant (Figure 1, step 10). Accordingly, the other positions are classified as IDPs, which cannot be mutated without radically altering the characteristics of the analyzed protein family, and VIPs, which are not good mutagenesis targets because they are "filling" amino acids or amino acids that contribute with indiscernible and different characteristics to each particular protein.

#### Identification of Invariable Determinant Positions

The identification of IDPs can be done much more easily than the identification of VDPs. Normally, IDPs can be directly identified by visual inspection and looking for complete or almost complete amino acid conservations at any given position. However, the identification of IDPs can be readily implemented as a part of the MOSST algorithm, using the same tools previously developed to identify VDPs. In fact, according to Figure 4, IDPs have two characteristic features: the variances of all the amino acid properties must have very low values (*i.e. *must be highly significant) and the differences between those variances must also be very small (*i.e. *differences are insignificant). Therefore, any position ***i ***for which the global probability ***P_i _***is classified as not significant and the probability ***p*_*i*,Ω,*n *_**for every **Ω **is classified as significant, must be an IDP (Figure 1, step 12).

#### Determination of the possibilities of mutation for each position

Once the amino acid positions in a protein whose mutation could be functionally significant have been identified, the possible effects of amino acid substitutions at such positions are determined in a further step of the MOSST algorithm. A distribution analysis of the different amino acid properties at said position is done in order to identify *a priori *possible functionally relevant or functionally silent amino acid changes in the protein, either to guide a rational mutagenesis strategy or to identify non-evident nsSNPs (Figure 1, step 11).

The proposed method determines the probability of an amino acid being present at a determined position using a statistical method based on the Student *t*-test. This procedure starts with the previously calculated average (***μ *_*i*,Ω,*n*_**) and variance estimator (***σ*_*i*,Ω,*n*_**) at a position ***i ***of the multiple alignment. For each position, each amino acid property is assumed to have a normal distribution ***N(μ,σ)***, with unknown parameters ***μ ***and ***σ***. This assumption is reasonable, because it is assumed that at each position the average of each scale would represent the ideal characteristic to be fulfilled at that position and the standard deviation would represent the accuracy level of conservation of the characteristic.

**Figure 7:**
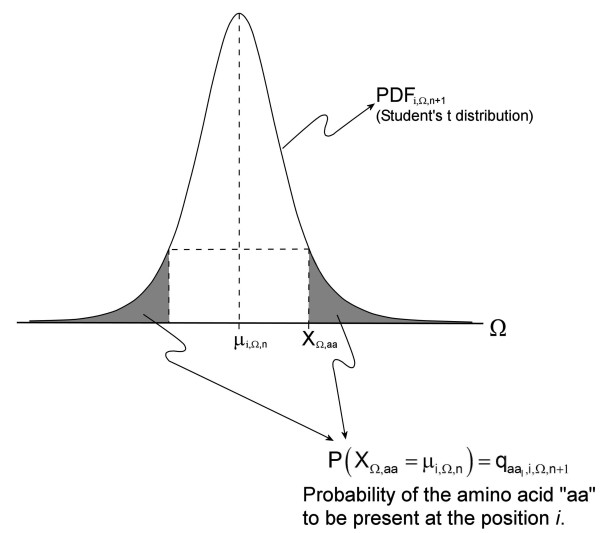
**Scheme depicting the posterior probability density function (PDF) of the values of a property Ω**. Continuing with the example of Figure 2, given the previously known group of ***n ***amino acids present in the multiple alignment at position ***i ***for a given amino acid **aa **having a value ***X*_Ω,aa _**assigned for the property **Ω**, and the average for **Ω **at position ***i ***being ***μ*_9,Ω,6_**, then the shadowed area represents the probability  that the amino acid **aa **could be present at the position ***i***, according to the property **Ω**.

For a sample of ***n ***values ***X*_*i*,Ω,*1*_...*X*_*i*,Ω,*n *_**obtained for an amino acid property **Ω **at a given position ***i***, assuming a normal distribution, it is possible to predict the distribution of a new value ***X*_*i*,Ω*,n+*1_**. Mathematically, if we have a normally-distributed variable ***X ***sample mean value ***μ*_*i*,Ω,*n *_**and a sample variance ***σ*_*i*,Ω,*n *_**for a position ***i***, then the test statistic:(4)

has a Student's t-distribution with ***n*-1 **degrees of freedom [57]. As the probability distribution of ***τ*_*i*,Ω,*n*+1 _**is known, it is possible to calculate the distribution of ***X*_*i*,Ω*,n+*1 _**as:(5)

 is the posterior cumulative distribution function for the values of the property **Ω **at position ***i***, given the previously known group of ***n ***amino acids present in the multiple alignment at such position. From this , a probability density function  can be derived. Since each amino acid naturally has one particular value of property **Ω**,  allows the assignment of a probability ***q***_***j,i***,Ω,***n***+1 _to each amino acid ***j ***(any of the 20 natural amino acids) to be present at position ***i ***in a new protein not included in the multiple alignment (see example in Figure 7). This probability is directly associated with the property **Ω **and its distribution .

As each amino acid can have many different properties **Ω_1_, Ω_2_... Ω*_L_***:, and each property will have a different PDF , then each amino acid will have ***L ***associated individual probabilities to be present at position ***i***, which we will call  for each amino acid ***j***. The combined global probability ***Q**_j,i _*for the occurrence of each amino acid ***j ***at position ***i ***is calculated as:(6)

This combined global probability represents the probability of every particular amino acid satisfying the requirements of all properties at position ***i***, and therefore is an indicator of the functional suitability of each amino acid in such position.

#### Quantification of the functional suitability of the amino acids

The former multi-property consensus method allows calculation of the global probability of occurrence of each amino acid at each position in a multiple alignment. The probability value can also be used as an indicator of the functional impairment introduced by each amino acid at every analyzed position ***i***. If the requirements of the functional properties that have to be fulfilled in position ***i ***lead to a high probability for an amino acid to be in that position, it means that the amino acid does not impair the function of the protein. Inversely, if the probability is low, such amino acid can be functionally deleterious.

**Figure 8:**
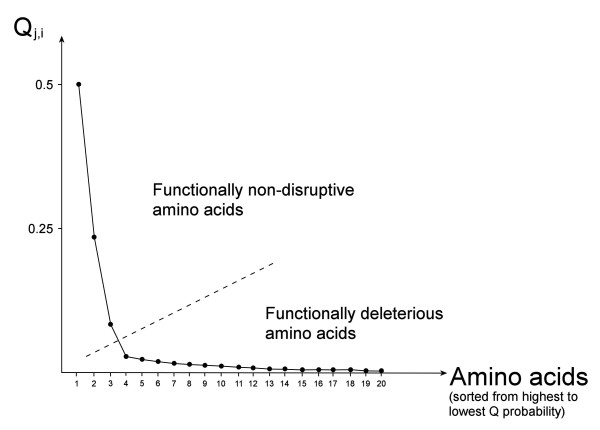
**Scree plot of the probabilities *Q_j,i _*of each amino acid being present at position *i***. The probabilities are sorted from highest (left) to lowest (right). A fall contrast or scree criterium is applied to this plot to identify a cut point in the curve (dashed line), where the highest probability factors are chosen up to a point where the curve becomes approximately horizontal.

For this last step of the analysis, a method based on the sorting of these probabilities of occurrence in descending order and the construction of a scree plot with the ordered values can be used. An example of this procedure is shown in Figure 8. The scree plot at each position ***i ***could help in getting the preference level or reliance with which the analyzed position could be occupied by each of the amino acids, based on the required physicochemical properties at such position in the protein. An analysis of the scree plot and its curvature can serve to identify a cut point (as indicated in Figure 8) in the curve. This point can be determined by using a "fall contrast" or "scree" criterium [58], where the highest probability factors are chosen up to a point where the curve becomes approximately horizontal. A second criterium can also be used, where the highest probability factors are chosen to explain together at least a predefined high probability (usually 95%) or until the last factor has a non-significant probability (usually less than 5%). Any criterium will define two sections in the scree plot: on the left a curve section including an amino acid group with the highest probabilities and on the right a section with those having the lowest probabilities. This classification allows separation of a high-probability group comprising functionally non-disruptive amino acids from a low-probability group of functionally deleterious amino acids. Hence, ***Q**_j,i _*can be used as a basis to identify non-evident nsSNPs and to design rational site-directed mutagenesis strategies for each position ***i ***in a protein.

### Implementation of the algorithm

Three main issues were solved to implement the algorithm. These aspects will not be analyzed in detail in this paper, because they are not directly related to the algorithm theory and design, although they must be solved to get a good implementation thereof.

The first aspect is the selection of suitable property vectors that could be successfully used in order to get the most accurate information from the MOSST algorithm and to exploit the classification abilities of the algorithm in an optimal way. Though the general algorithm has been presented as being able to use any group of property vectors to perform the proposed analysis, we selected a minimal number of three normalized vectors that are mathematically orthogonal to each other to optimize the statistical potency of the results.

The second issue is the calculation of the probability distributions of the averages and variances of the selected optimal vectors, which are necessary for the implementation of the algorithm as set forth in the present paper. We have assessed that the Law of Large Numbers is not accurately applicable for combinations of less than 30 amino acids given the optimal vectors previously obtained. Even though the probabilities of the different amino acid combinations could be calculated in a direct way, it is computationally difficult and time-consuming for combinations of more than 5 or 6 amino acids. We developed a direct calculation method to get an approximate distribution for combinations from 2 to 30 amino acids, which analysis and explanation is out of the scope of this work.

The last aspect is the determination of the right significance levels that should be taken into account to classify each position as "non significant", "significant" and "very significant" when determining the rejection regions for the null hypothesis of equality of variances. As we mentioned earlier, values of 0.01 and 0.005 could be preliminary used to select between these classifications, which are more stringent than those values usually used in statistical testing. However, our experience implementing the algorithm has shown that the most interesting positions in a protein alignment (which we called "primary positions") have to be determined case by case, based on characteristics of each different multiple alignment. In our experience, the implementation of the algorithm included this data analysis in order to get the most out of the results of the general algorithm.

The MOSST algorithm has been implemented with the details mentioned above as a Graphical User Interface (GUI) in MATLAB (see Additional File 1, Additional File 2 and Additional File 3). Given the nature of MOSST results, it is easier to display them in a graphical interface in order to allow the user to have an integral vision over them and to modify the parameters of the algorithm on-the-run to obtain the best possible results for each given analysis. One of the major advantages we devised from the use of an interactive GUI is the definition of "primary positions". We defined primary positions as those "very significant" positions that have exceptionally high mutability scores as defined by the statistic theory behind the algorithm. By using MOSST GUI, the user can select both manually or semi-automatically the threshold significance value above which a "very significant" position becomes a "primary position" and thus a primary mutagenesis objective.

The semi-automatic method advantage over the manual method is that it helps the user to select a suitable threshold by presenting a differential scree plot where the user can interactively select the threshold by comparing the differences (step sizes) between similar ordered significance values. The optimum threshold will be a "very significant" significance value having the largest possible difference with immediately higher (adjacent) significance values. If two or more significance values fulfill these criteria, the largest one is most appropriately selected. This empirical rule is applied by the user with his/her own judgment to select a proper threshold value.

The empirical selection of "primary positions" proved to be an additional tool to narrow the search for mutable positions in a protein family. An example of the relevance of determining primary positions is shown in the study of glycosyl hydrolases belonging to family 16.

### Testing: mutagenesis analysis of endoglucanases belonging to family 16 of the glycosyl hydrolases

#### Selection of representative proteins

MOSST was used for the analysis of representative endoglucanases classified as glycosyl hydrolases belonging to family 16. They have several appropriate characteristics: (1) a very close structural and folding similarity despite them not having a very high degree of sequence similarity, (2) well characterized residues that participate in the interaction with the substrates at the active site and define the substrate specificity of the family, (3) well characterized active residues that are indispensable for catalysis, (4) two well defined and populated enzyme families with distinct substrate specificities and no known structure-function correlations, namely lichenases (endo-1,3-1,4-β-D-glucanases, EC 3.2.1.73) and laminarinases (endo-1,3-β-D-glucanases, EC 3.2.1.39, endo-1,3(4)-β-D-glucanases, EC 3.2.1.6), and (5) large availability of experimental data, including mutagenic studies. These characteristics were considered ideal to test the ability of MOSST to determine functionally relevant amino acid positions against experimentally tested results, since this allows the analysis of amino acid mutations purely by the effect of their side groups, while leaving other factors (molecular structure, catalytic properties, loop variations) outside of the analysis.

Among these enzymes, we selected *Cellulosimicrobium cellulans*'s BglII as our reference enzyme. Proteins included in the analysis were selected by their similarity with BglII and both the conservation of folding pattern and enzymatic activity. Table 2 shows the representative set of proteins selected and included in this study.

**Table 2:** Analyzed endoglucanases belonging to glycosyl hydrolases family 16

#	Swiss Prot code	EMBL or GenBank code	Description	Organism	Enzymatic classification
1	O68641	**AF052745**	β-1,3-glucanase II (BglII)	*Cellulosimicrobium cellulans*	Laminarinase
2	Q51333	**U56935**	β-1,3-glucanase IIa (BglIIa)	*C. cellulans*	Laminarinase
3	Q60039	**Z47974**	Laminarinase	*Thermotoga neapolitana*	Laminarinase (EC 3.2.1.39)
4	Q9WXN1	**AE001690**	Laminarinase	*Thermotoga maritima*	Laminarinase
5	O73951	**AF013169**	endo-β-1,3-glucanase (precursor)	*Pyrococcus furiosus*	Putative laminarinase
6	O52754	**AF047003**	Laminarinase	*Rhodothermus marinus*	Laminarinase
7	**P45798**	U04836	β-glucanase (precursor)	*R. marinus*	Lichenase (EC 3.2.1.73)
8	Q45095	**JN0772**	β-1,3-glucanase bglH (precursor)	*Bacillus circulans*	Putative lichenase
9	**P23903**	M34503	Glucan endo-1,3-β-glucosidase A1 (precursor)	*B. circulans*	Laminarinase (EC 3.2.1.39)
10	Q9Z3Q2	**AJ225896**	endo-1,3-1,4-β-glucanase eglC	*Rhizobium meliloti*	Putative lichenase (EC 3.2.1.-)
11	O33680	**U89164**	endo-1,3-1,4-β-glucanase exsH	*R. meliloti*	Putative lichenase (EC 3.2.1.-)
12	**P07980**	M15674	β-glucanase (precursor)	*Bacillus amyloliquefaciens*	Lichenase (EC 3.2.1.73)
13	Q45691	**U60830**	endo-β-1,3-1,4-glucanase	*Bacillus subtilis*	Putative lichenase
14	P04957	**X00754**	β-glucanase (precursor)	*B. subtilis*	Lichenase (EC 3.2.1.73)
15	-	CAA81096 (**Z25877**)	hybrid endo-1,3-1,4-β-glucanase (synthetic construct)	*Bacillus macerans */ *B. amyloliquefaciens*	Putative lichenase
16	**P27051**	X57279	β-glucanase (precursor)	*Bacillus licheniformis*	Lichenase (EC 3.2.1.73)
17	-	CAA81092 (**Z25873**)	hybrid endo-1,3-1,4-β-glucanase (synthetic construct)	*B. macerans */ *B. amyloliquefaciens*	Putative lichenase
18	-	CAA81094 (**Z25875**)	hybrid endo-1,3-1,4-β-glucanase (synthetic construct)	*B. macerans */ *B. amyloliquefaciens*	Putative lichenase
19	**P45797**	X57094	β-glucanase (precursor)	*Paenibacillus polymyxa*	Lichenase (EC 3.2.1.73)
20	P29716	**X58392**	β-glucanase (precursor)	*Clostridium thermocellum*	Lichenase (EC 3.2.1.73)
21	P29716	X63355 **(JS0611)**	β-glucanase (precursor)	*C. thermocellum*	Lichenase (EC 3.2.1.73)
22	O14412	**U63813**	β-glucanase (precursor)	*Orpinomyces sp*. PC-2	Lichenase (EC 3.2.1.73)
23	P37073	M84339 **(A48378)**	β-glucanase (precursor)	*Bacillus brevis*	Lichenase (EC 3.2.1.73)
24	Q59328	**X89732**	Endo-1,3(4)-β-glucanase	*C. thermocellum*	Laminarinase (EC 3.2.1.6)
25	Q26660	**U49711**	β-1,3-glucanase	*Strongylocentrotus purpuratus*	Putative laminarinase
26	-	**U42580** (AAC96462)	β-1,3-glucanase	*Paramecium bursaria* *chlorella virus *(PBCV-1)	Putative laminarinase

Descriptor codes in the multiple alignment are highlighted in bold characters.

#### Obtention of a multiple alignment

Following the flow diagram of the MOSST algorithm, these proteins were aligned using ClustalW (http://www.ebi.ac.uk/clustalw/, [12]). Preliminary tests using different substitution matrices and different gap opening and extension penalties were performed. The combination that exhibited best results was obtained using an identity matrix as substitution matrix and minimal values for gap creation and extension. This combination empirically gives more importance to conserved sectors, which in our case correspond to secondary structure and folding sectors that are conserved in the family. This strategy is concordant with the fact that the studied group of proteins is a very structurally conserved enzyme family, even when conservation is lower at the sequence level due to the difference in activities of the proteins.

#### Redundancy analysis

**Figure 9:**
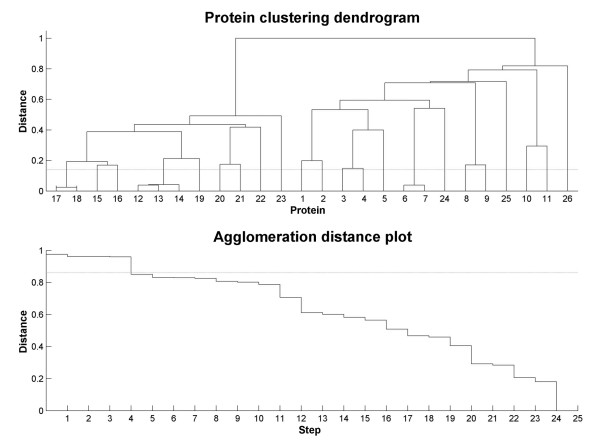
**Auxiliary plots for redundancy removal in the analyzed protein family**. The top dendrogram shows the clustering of proteins according to the distance (similarity percentage) between them. The bottom plot is an agglomeration distance plot of the top dendrogram. In both plots a horizontal line representing a similarity percentage of 86% that was taken as the limit over which two proteins were considered as identical. This value is the minimal value within the most pronounced step in the agglomeration distance plot. The numbers of the different proteins are the order numbers assigned in Table 2.

For this objective, a protein clustering dendrogram and an agglomeration plot were constructed according to the similarity percentage between the proteins in the multiple alignment (Figure 9). An optimal separation between sub-families is obtained using a similarity percentage cutoff of 86%. Three groups of similar enzymes were represented by only one of them: (i) synthetic hybrid lichenases 17 and 18 from *Bacillus macerans */ *Bacillus amyloliquefaciens *(enzyme numbers are given with reference to the order numbering in Table 2), (ii) *Bacillus amyloliquefaciens *(12) and *Bacillus subtilis *(13 and 14) lichenases, and (iii) two enzymes from *Rhodothermus marinus*, one of them classified as a laminarinase (6) and the other classified as a lichenase (7). The remaining proteins, even those belonging to the same species, are considered different enough to be included in the analysis with no added redundancy.

It can be observed in the dendrogram a subdivision of the proteins in the alignment in two large sub-families corresponding mainly to the division between lichenases (left-hand sub-family in the dendrogram) and laminarinases (right-hand sub-family). This observation indicates that the information contained in the multiple alignment is also able to discriminate between functional aspects of the enzymes.

#### Calculation of the statistic values and significances

For each of the positions of the non-redundant multiple alignment, the associated variance in each of the three principal components and the differences between the three components were calculated. The values of said differences were used to calculate the global significance of each position and to classify said significance according to the criteria exposed in the previous paper of this series. This allowed the classification of positions in the alignment as "non-significant", "significant" and "very significant". In addition, a new category of "primary position" was defined when the global significance of the position is included in the higher percentile of the values in the distribution of global variances as described in the practical implementation of MOSST analysis.

**Figure 10:**
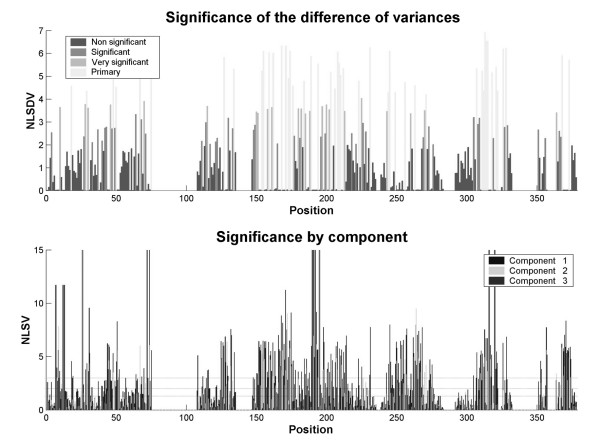
**Result plots for the global significance of the positions (top) and the significances of variances in each component (bottom)**. NLSDV: negative of the base-10 logarithm of the significance of the difference of variances; NLSV: negative of the base-10 logarithm of the significance of variances.

Figure 10 shows the graphical results obtained for variance probabilities for each component and each position, and the results obtained for global significances at each position. In these plots, many positions can be identified as "primary" mutagenesis targets as defined before and also many "very significant" positions, all of them scattered along the catalytic domain of endoglucanases belonging to glycosyl hydrolases family 16. However, the distribution of primary mutagenesis targets is not uniform, but said targets are concentrated in more or less defined sectors in the amino acid sequence of the protein, which gives a hint about the existence of determinant and non-determinant regions for this enzyme family.

#### Structural mapping of primary positions

**Figure 11:**
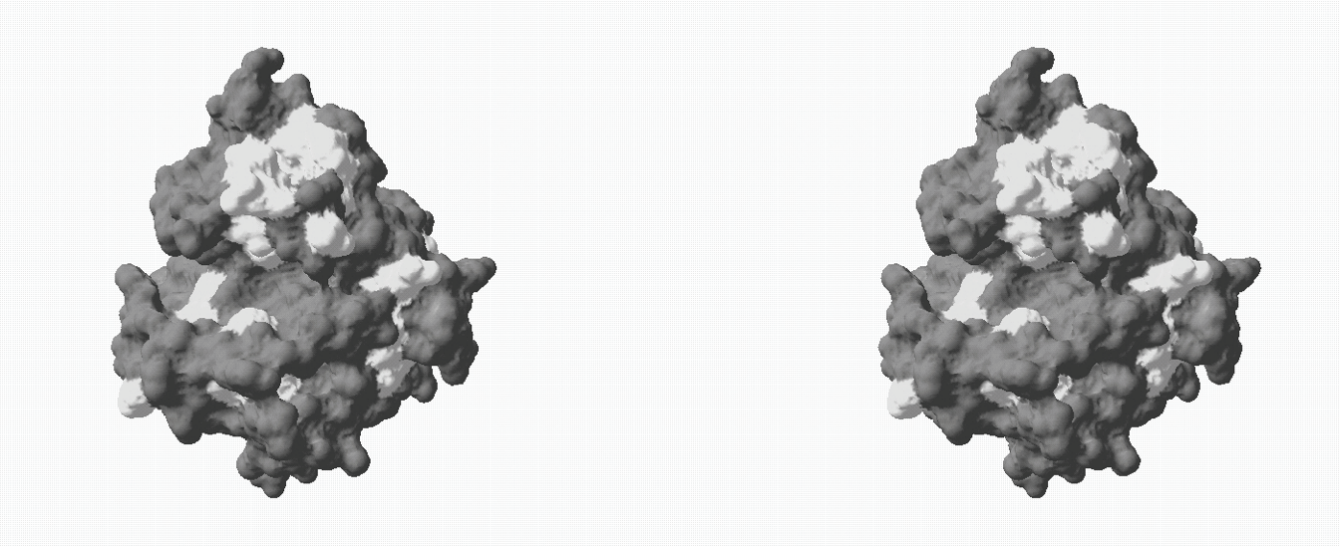
**3D mapping of the amino acids onto the 3D structure of BglII**. Mutagenically interesting positions (light grey) are mapped over the molecular structure of the catalytic domain of BglII, selected as a representative structure of family 16 glycosyl hydrolases (order number 1 in Table 2). This figure is a cross-eyed stereogram.

**Figure 12:**
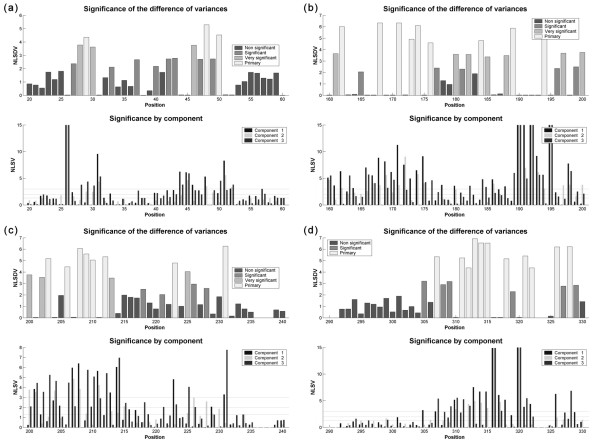
**Result plots for the amino acids that form the active site of BglII**. Global significance of positions and significances of variances for each component, for positions in the multiple alignment corresponding to amino acids that form the active site of BglII (and other family 16 glycosyl hydrolases): (a) positions 20-60; (b) positions 160-200; (c) positions 200-240; and (d) positions 290-330.

The determined primary positions can be mapped over a model of the tridimensional structure of BglII. In this structural mapping a spatial distribution pattern of sectors in which variable determinant positions are concentrated can be observed, i.e. the most promising mutagenesis targets for this protein family (Figure 11). The active site of the protein family is comprised widely by amino acids in positions 45-49 (Asn26-Gln30), 167-179 (Ile89-Ser99), 187-196 (Ser107-Asn116), 205-210 (His125-Gly130), 214-222 (Gly132-Ile139), 307-311 (Phe191-Phe195) and 315-319 (Leu199-Val203) in the multiple alignment. A detailed analysis of active site amino acids according to the algorithm implemented in this work is shown in Figure 12. Inside the active site, variable determinant positions were identified as the following amino acids using BglII numbering (and their position in the multiple alignment indicated between brackets): Leu29 (48), Trp90 (168), Phe93 (171), Met95 (173), Leu96 (174), Gly109 (189), Met114 (194), Gly126 (206), Val128 (208), His129 (209), Gly130 (210), Phe191 (307), Phe195 (311), Leu199 (315) and Ala202 (318).

#### Predictive value of the algorithm: comparative analysis of laminarinases

**Figure 13:**
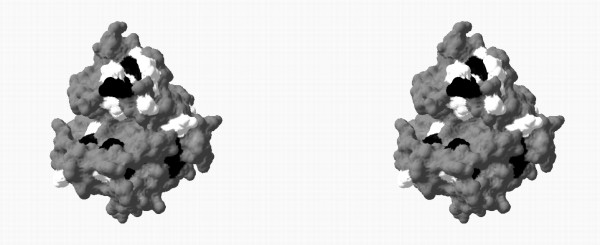
**Comparative mapping of primary variable determinant positions**. Primary variable determinant positions for all the studied protein family (lichenases and laminarinases) are shown in white and black. White positions are variable determinant amino acids both for lichenases and laminarinases, while black positions are primary variable determinant positions specific for laminarinases. This figure is a cross-eyed stereogram.

The MOSST algorithm can be applied only to an enzyme sub-family. In our case, these sub-families can be determined from the redundancy removal dendrogram and are associated with enzyme function differences, as mentioned earlier. It is interesting to apply this analysis procedure to laminarinases and compare these results with the variable determinant positions obtained for the entire family. Therefore, variable determinant positions were determined for laminarinases using a similar procedure to that used for the total protein family, and these positions were compared with those obtained for all family 16 glucanases. The active site structure of BglII is represented in Figure 13 with differentiated variable determinant amino acids for family 16 glucanases and variable determinant positions for the laminarinase sub-family. Logically, if a position in the active site is variable for laminarinases as a family then said position cannot be significant to determine substrate specificity of these enzymes. Instead, if a position is variable determinant for family 16 glucanases but not for laminarinases, then it is logical to assume that said position could be relevant in the determination of laminarinase specificity when compared with the other enzymes of the same family.

Under this assumption, the comparative analysis using the MOSST algorithm shows that the following active site amino acids (having either exposed or buried side chains) can have a determinant function over laminarinase specificity: Leu29, Trp90, Gly109, Gly130, Phe191, Phe195 and Ala202. It is interesting to asses that some of these amino acids have been identified in the analysis of the molecular model of BglII and other family 16 glucanases as amino acids that participate in substrate binding and/or interactions [59,60]. This observation corroborates the ability of the MOSST algorithm to identify relevant positions in a protein.

#### Predictive value of the algorithm: analysis of histidine 129

Position 209 (Figure 12), which corresponds to histidine 129 in BglII, is very remarkable. This position has been identified in the structural analysis of BglII as an amino acid that could be implied in the chemical reactions catalyzed by this enzyme [61].

The MOSST algorithm marks position 209, i.e. histidine 129, in BglII as a primary mutagenesis objective and therefore this fact can be exploited to obtain interesting variants of the enzyme, possibly having different catalytic properties due to the position occupied by said amino acid in the active site. It should be noted that this histidine is classified as a primary amino acid both for the global family of proteins under study and for laminarinases, and so a possible substrate interaction function should be discarded, at least in a first approach.

MOSST algorithm suggests for said position the presence of a histidine residue (with a 93.5% of probability) and the alternative presence of a phenylalanine residue (2.4% probability) or a glutamine residue (1.5% probability). The remaining amino acid have probabilities of less than 1% each and their combined probabilities only adds to 2.6%, so their presence is not considered relevant in said position. The real nature and contribution of each proposed alternative could be analyzed using other predictive techniques, in advance to mutagenic experiments, in order to decide which of said variants would be the most favorable one to obtain the desired effects in the protein. It is also possible to analyze all the proposed alternatives, producing in the laboratory the corresponding mutant enzymes and analyzing their new properties.

The contribution of this amino acid and the possible mutations performed on it to the enzymatic catalysis could be assessed by means of an additional analysis, for example, a kinetic analysis of a catalytic model. In a former work [61], using structural and mechanistic knowledge about glycosyl hydrolases from families 7, 10, and 16, we have formulated a mathematical model that can include ionizable residues in the active site and incorporating electrostatic influences via acid dissociation equilibrium constants and chemical relationships such as hydrogen bonds. The results of the simulations indicated a clear shift in the pH dependence of activity for the enzymes only when a close interrelation (hydrogen bond) between a catalytic glutamate and histidine 129 is taken into account, which is concordant with experimental evidence with BglII (manuscript in preparation). Moreover, we demonstrated that the presence of the kind of chemical interaction proposed could provide stabilization of the activity in the presence of environmental, structural, pH and electrostatic variations. The results suggested a new way to modify, via site-directed mutagenesis, the acid dissociation of one of the catalytic residues in the active site independently of the other, which could have clear advantages over the purely electrostatic modifications that usually affect both residues simultaneously.

Under the light of this previous work, MOSST analysis results were very striking in their ability to identify this residue as relevant even without any previous knowledge or suspicion about its relevance or function in this enzyme family. In our opinion, this clearly demonstrates the usefulness of MOSST as a general tool for the design of new site-directed mutagenesis strategies in protein families.

## Conclusions

A statistical procedure has been designed and presented to semi-automatically identify functionally significant mutable positions in a protein, based on the conservation of physicochemical properties. Such positions are identified and classified into three groups, according to the influence their mutation could have on protein function. Those in which a mutation does not alter the function and basic characteristics of the protein, but do change them slightly, and those in which a mutation is totally deleterious for the protein are the most relevant positions to look for nsSNPs, while only the former are important when trying to develop site-directed mutagenesis strategies so that variants with improved properties could be generated.

Amino acid positions are also classified in three groups: variable irrelevant positions (VIPs), invariable determinant positions (IDPs) and variable determinant positions (VDPs). The recognition and identification of VDPs is especially relevant, since these are "hidden" functionally relevant positions that cannot be easily identified by other existing automatic methods. This classification serves as a basis to rationally identify relevant amino acid positions in a protein in the frame of a rational design strategy or in the identification of nsSNPs. In this way, a conservative mutagenesis strategy that aims to preserve the main properties of a protein family should be focused on mutating only VDPs, while a non-conservative mutagenesis strategy could also include IDPs. If the aim is to identify nsSNPs, mutations that could alter protein function are most probably located at IDPs and VDPs.

Once these key functional positions are identified, the statistical distribution of the relevant physicochemical properties at each protein position is analyzed to get a list of the 20 common protein amino acids ordered according to the global probability with which they can conform to the required property profile of each relevant position. This ordered list is divided into a group of functionally non-disruptive amino acids and a second group of functionally deleterious amino acids.

These results become a discriminative tool in the selection and elaboration of rational mutagenesis strategies for the protein. They can also be used to predict if a given nsSNP, identified, for instance, in a genomic-scale analysis, can have a functional implication for a particular protein and which nsSNPs are most likely to be functionally silent for a protein. This analytical tool could be used to rapidly discard any irrelevant nsSNP and guide the research focus toward functionally significant mutations. This approach also has the advantage that not only conserved amino acid positions in a protein family can be labeled as functionally relevant, but also non-conserved amino acid positions can be identified having a functional effect.

The MOSST algorithm has been implemented as a MATLAB GUI and used to analyze endoglucanases belonging to glycosyl hydrolases family 16, in order to determine interesting mutagenesis targets on them. The analysis has indicated amino acids that could be mutated in β-1,3-endoglucanase BglII of *C. cellulans *to obtain critical changes in the enzymatic activity: putative substrate specificity amino acid determinants for lichenase and laminarinase activity and determinants of pH-activity profiles for these enzyme family, thus confirming MOSST performance as a predictive tool for the study of functionally relevant mutations.

The proposed methodology also has limitations, especially in that it uses only information derived from a multiple alignment and the statistical result for each position will strongly depend on the quality of the alignment and it does not prevent *a priori *this significance level to vary between one alignment and another. In this sense, the quality of the alignment is determinant and should be carefully controlled. Moreover, the algorithm does not give any suggestion about the real nature and contribution of relevant amino acids to the structure and function of the protein, but the predictions could be analyzed using other predictive techniques or tested by mutagenesis experiments. Another limitation is related to the fact that MOSST operates on single positions in a multiple alignment and all statistic parameters are calculated for each position without taking into consideration the simultaneous occurrence of mutations in other positions that can compensate for the effect of the first. This excludes the study of correlated compensatory mutations using MOSST in its current version.

Although the developed procedure does not give any indication about the functional implications of the amino acids positions identified as relevant, an unrelated analysis that use another type of information beyond the statistical inference performed on the multiple alignment by MOSST could shed light on their contribution to the protein function or structure. Specifically, classical methods to study experimentally and/or predictively the structure-function relationships in proteins can provide this kind of external information. For example, this type of analysis has been used to test the functional relevance of the amino acids identified by MOSST in family 16 glycosyl hydrolases. In the case of position 209 of the alignment of these proteins, a mechanistic-electrostatic analysis yielded an explanation of the functional contribution of this amino acid position to catalysis [61]. The implementation and testing results set forth in this work show a promising performance of this technique as a valuable bioinformatics tool to aid in the development of new protein variants and to aid in the understanding of function-structure relationships in proteins.

## Methods

### Determination of optimal classification properties for MOSST

55 properties measured by scales that are used to rate and sort amino acids were selected from the literature, including the most frequently used scales of hydrophobicity or hydrophilicity and secondary structure conformational scales, as well as many others based on different chemical and physical properties of amino acids.

To find the underlying organization in this varied group of characteristics and remove data redundancy, property scales were normalized and a clustering analysis was performed on them. The analysis indicated that it is not necessary to use a larger number of scales to obtain a more accurate classification of the natural amino acids. The clustering analyses performed classified the 55 amino acid scales considered in 7 clusters of variables, each cluster sharing similar characteristics and tendencies and representing a defined physicochemical property.

To obtain a set of independent (orthogonal) variables, a principal component analysis was performed over the seven vectors obtained from the cluster analysis. Three principal component vectors were extracted from the set of seven vectors that resulted from the cluster analysis, these three factors representing 94.1% of the variance within said vectors and normalized. A physical representation was assigned to each of these factors using correlation analyses with the original variables. An amino acid classification test was performed using these three orthogonal properties, which agrees with the practical biochemical experience (data not shown).

### Variance and average distributions for each orthogonal property

The cumulative distribution functions (CDFs) of the variances calculated for the population of random combinations of ***n ***amino acids were determined for each extracted property, calculating the average and standard deviation for all the possible amino acid combinations of ***n *= 2 ... 30 **using a recursive discretization algorithm. The distributions for combinations of higher numbers of amino acids was estimated as equal to the combination of 30 amino acids, following the Law of Large Numbers. A similar approach was used to calculate the CDFs and PDFs for combinations of ***n ***amino acids. The probability distribution function of the differences of variances between any two components was calculated following the same methodological approach employed in the calculation of the PDFs for the variances of different amino acid combinations.

### Practical implementation of MOSST algorithm

MOSST results were implemented in a graphical interface in order to allow the user to have an integral vision over them and to modify the parameters of the algorithm on-the-run to obtain the best possible results for each given analysis. In order to achieve this goal, the MOSST algorithm was implemented using MATLAB Graphical User Interface (GUI). MATLAB also provided the mathematical routines to allow a fast and robust mathematical treatment of the data and results.

The use of this GUI allows manual determination of "primary positions", i.e. those "very significant" positions that have exceptionally high mutability scores as defined by the statistic theory behind the algorithm. By using the MOSST graphical interface, the user can select both manually or semi-automatically the threshold significance value above which a "very significant" position becomes a "primary position" and thus a primary mutagenesis objective.

Additional File 1 annexed to this manuscript includes general instructions to install and use the MATLAB GUI implementation of MOSST, Additional File 2 contains all routines for the MATLAB GUI implementation of MOSST and exemplary alignment files for family 16 glycosyl hydrolases, and Additional File 3 is a basic user guide to run and operate the MOSST MATLAB GUI implementation.

## Authors' contributions

AO-N designed the MOSST algorithm, developed the statistical and mathematical methods of this study, defined the biologically meaningful cases and classifications used by the algorithm to predict rational protein design strategies and identify nsSNPs, implemented the algorithm as a MATLAB GUI and tested the algorithm performance on family 16 of the glycosyl hydrolases. BAA helped to define the biochemical background used by the algorithm and participated in refining the methodology followed by the algorithm, defining the natural applications of the algorithm in rational protein design and identification of nsSNPs, and interpreting the results delivered by the algorithm. JAA helped to conceive and design the biotechnological use of this algorithm and to refine the methodology followed by the algorithm, provided the example applications to test the algorithm performance and coordinated this study. All authors contributed to draft the manuscript, and read and approved the final version.

## Supplementary Material

Additional file 1 **README FIRST!.pdf (Portable Document Format)**; file with general information and user instructions. Click here for file

Additional file 2 **MOSST Essential Files.zip (ZIP format)**; compressed file including all routines for the MATLAB GUI implementation of MOSST and exemplary alignment files for family 16 glycosyl hydrolases. Click here for file

Additional file 3 **Basic User Guide.pdf (Portable Document Format)**; user guide with short explanations and instructions to run and operate the MOSST MATLAB GUI implementation.
These additional files and MOSST latest developments can be found at the website of the Millennium Institute for Cell Dynamics and Biotechnology (ICDB) http://www.icdb.cl. Click here for file

## References

[B1] Atchley WR Wollenberg KR Fitch WM Terhalle W Dress AW Correlations among amino acid sites in bHLH protein domains: an information theoretic analysis Mol Biol Evol 2000 17 164 178 10666716 10.1093/oxfordjournals.molbev.a026229

[B2] Koshi JM Goldstein RA Mutation matrices and physical-chemical properties: correlations and implications Proteins 1997 27 336 344 10.1002/(SICI)1097-0134(199703)27:3<336::AID-PROT2>3.0.CO;2-B 9094736

[B3] Kono H Wang W Saven JG Arndt KM, Müller KM Combinatorial protein design strategies using computational methods Protein engineering protocols 2006 352 Totowa, New Jersey: Humana Press 3 22

[B4] Li B Gallin WJ Computational identification of residues that modulate voltage sensitivity of voltage-gated potassium channels BMC Struct Biol 2005 5 16 10.1186/1472-6807-5-16 16111489 PMC1208917

[B5] Casari G Sander C Valencia A A method to predict functional residues in proteins Nat Struct Biol 1995 2 171 178 10.1038/nsb0295-171 7749921

[B6] Mirny LA Gelfand MS Using orthologous and paralogous proteins to identify specificity-determining residues in bacterial transcription factors J Mol Biol 2002 321 7 20 10.1016/S0022-2836(02)00587-9 12139929

[B7] Ng PC Henikoff S Predicting deleterious amino acid substitutions Genome Res 2001 11 863 874 10.1101/gr.176601 11337480 PMC311071

[B8] Ng PC Henikoff S SIFT: Predicting amino acid changes that affect protein function Nucleic Acids Res 2003 31 3812 3814 10.1093/nar/gkg509 12824425 PMC168916

[B9] Bao L Cui Y Prediction of the phenotypic effects of non-synonymous single nucleotide polymorphisms using structural and evolutionary information Bioinformatics 2005 21 2185 2190 10.1093/bioinformatics/bti365 15746281

[B10] Bhinge A Chakrabarti P Uthanumallian K Bajaj K Chakraborty K Varadarajan R Accurate detection of protein:ligand binding sites using molecular dynamics simulations Structure 2004 12 1989 1999 10.1016/j.str.2004.09.005 15530363

[B11] Kato R Nakano H Konishi H Kato K Koga Y Yamane T Kobayashi T Honda H Novel strategy for protein exploration: high-throughput screening assisted with fuzzy neural network J Mol Biol 2005 351 683 692 10.1016/j.jmb.2005.05.026 16019025

[B12] Thompson JD Higgins DG Gibson TJ CLUSTAL W: improving the sensitivity of progressive multiple sequence alignment through sequence weighting, position-specific gap penalties and weight matrix choice Nucleic Acids Res 1994 22 4673 4680 10.1093/nar/22.22.4673 7984417 PMC308517

[B13] Henikoff S Henikoff JG Amino acid substitution matrices from protein blocks Proc Natl Acad Sci USA 1992 89 10915 10919 10.1073/pnas.89.22.10915 1438297 PMC50453

[B14] Gonnet GH Cohen MA Benner SA Exhaustive matching of the entire protein sequence database Science 1992 256 1443 1445 10.1126/science.1604319 1604319

[B15] Aboderin A An empirical hydrophobicity scale for α-amino-acids and some of its applications Int J Biochem 1971 2 537 544 10.1016/0020-711X(71)90023-1

[B16] Abraham DJ Leo AJ Extension of the fragment method to calculate amino acid zwitterion and side chain partition coefficients Proteins 1987 2 130 152 10.1002/prot.340020207 3447171

[B17] Bairoch A Release notes for SWISS-PROT. Release 38. July 1999 1999 Geneva

[B18] Bhaskaran R Ponnuswamy PK Positional flexibilities of amino acid residues in globular proteins Int J Pept Protein Res 1988 32 242 255 10.1111/j.1399-3011.1984.tb00944.x 6480218

[B19] Black SD Mould DR Development of hydrophobicity parameters to analyze proteins which bear post- or cotranslational modifications Anal Biochem 1991 193 72 82 10.1016/0003-2697(91)90045-U 2042744

[B20] Browne CA Bennett HP Solomon S The isolation of peptides by high-performance liquid chromatography using predicted elution positions Anal Biochem 1982 124 201 208 10.1016/0003-2697(82)90238-X 7125223

[B21] Bull HB Breese K Surface tension of amino acid solutions: a hydrophobicity scale of the amino acid residues Arch Biochem Biophys 1974 161 665 670 10.1016/0003-9861(74)90352-X 4839053

[B22] Chothia C The nature of the accessible and buried surfaces in proteins J Mol Biol 1976 105 1 12 10.1016/0022-2836(76)90191-1 994183

[B23] Chou PY Fasman GD Prediction of the secondary structure of proteins from their amino acid sequence Adv Enzymol Relat Areas Mol Biol 1978 47 45 148 364941 10.1002/9780470122921.ch2

[B24] Cowan R Whittaker RG Hydrophobicity indices for amino acid residues as determined by high-performance liquid chromatography Pept Res 1990 3 75 80 2134053

[B25] Deleage G Roux B An algorithm for protein secondary structure prediction based on class prediction Protein Eng 1987 1 289 294 10.1093/protein/1.4.289 3508279

[B26] Eisenberg D Schwarz E Komaromy M Wall R Analysis of membrane and surface protein sequences with the hydrophobic moment plot J Mol Biol 1984 179 125 142 10.1016/0022-2836(84)90309-7 6502707

[B27] Fauchère J-L Pliska V Hydrophobic parameters pi of amino-acid side chains from the partitioning of N-acetyl-amino-acid amides Eur J Med Chem - Chim Ther 1983 18 369 375

[B28] Fraga S Theoretical prediction of protein antigenic determinants from amino acid sequences Can J Chem 1982 60 2606 2610 10.1139/v82-374

[B29] Grantham R Amino acid difference formula to help explain protein evolution Science 1974 185 862 864 10.1126/science.185.4154.862 4843792

[B30] Guy HR Amino acid side-chain partition energies and distribution of residues in soluble proteins Biophys J 1985 47 61 70 10.1016/S0006-3495(85)83877-7 3978191 PMC1435068

[B31] Hopp TP Woods KR Prediction of protein antigenic determinants from amino acid sequences Proc Natl Acad Sci USA 1981 78 3824 3828 10.1073/pnas.78.6.3824 6167991 PMC319665

[B32] Janin J Surface and inside volumes in globular proteins Nature 1979 277 491 492 10.1038/277491a0 763335

[B33] Jones DD Amino acid properties and side-chain orientation in proteins: a cross correlation approach J Theor Biol 1975 50 167 183 10.1016/0022-5193(75)90031-4 1127956

[B34] Kyte J Doolittle RF A simple method for displaying the hydropathic character of a protein J Mol Biol 1982 157 105 132 10.1016/0022-2836(82)90515-0 7108955

[B35] Levitt M Conformational preferences of amino acids in globular proteins Biochemistry 1978 17 4277 4285 10.1021/bi00613a026 708713

[B36] Lifson S Sander C Antiparallel and parallel beta-strands differ in amino acid residue preferences Nature 1979 282 109 111 10.1038/282109a0 503185

[B37] Manavalan P Ponnuswamy PK Hydrophobic character of amino acid residues in globular proteins Nature 1978 275 673 674 10.1038/275673a0 703834

[B38] McCaldon P Argos P Oligopeptide biases in protein sequences and their use in predicting protein coding regions in nucleotide sequences Proteins 1988 4 99 122 10.1002/prot.340040204 3227018

[B39] Meek JL Prediction of peptide retention times in high-pressure liquid chromatography on the basis of amino acid composition Proc Natl Acad Sci USA 1980 77 1632 1636 10.1073/pnas.77.3.1632 6929513 PMC348551

[B40] Miyazawa S Jernigan R Estimation of effective interresidue contact energies from protein crystal structures: quasi-chemical approximation Macromolecules 1985 18 534 552 10.1021/ma00145a039

[B41] Mohana JK Rao Argos P A conformational preference parameter to predict helices in integral membrane proteins Biochim Biophys Acta 1986 869 197 214 10.1016/0167-4838(86)90295-5 2935194

[B42] National Biomedical Research Foundation, Dayhoff MO Protein segment dictionary 78: from the Atlas of protein sequence and structure, volume 5, and supplements 1, 2, and 3 1978 Silver Spring MD, Washington D.C.: National Biomedical Research Foundation, Georgetown University Medical Center

[B43] Parker JM Guo D Hodges RS New hydrophilicity scale derived from high-performance liquid chromatography peptide retention data: correlation of predicted surface residues with antigenicity and X-ray-derived accessible sites Biochemistry 1986 25 5425 5432 10.1021/bi00367a013 2430611

[B44] Rose GD Geselowitz AR Lesser GJ Lee RH Zehfus MH Hydrophobicity of amino acid residues in globular proteins Science 1985 229 834 838 10.1126/science.4023714 4023714

[B45] Roseman MA Hydrophilicity of polar amino acid side-chains is markedly reduced by flanking peptide bonds J Mol Biol 1988 200 513 522 10.1016/0022-2836(88)90540-2 3398047

[B46] Sweet RM Eisenberg D Correlation of sequence hydrophobicities measures similarity in three-dimensional protein structure J Mol Biol 1983 171 479 488 10.1016/0022-2836(83)90041-4 6663622

[B47] Welling GW Weijer WJ van der Zee R Welling-Wester S Prediction of sequential antigenic regions in proteins FEBS Lett 1985 188 215 218 10.1016/0014-5793(85)80374-4 2411595

[B48] Wilson KJ Honegger A Stotzel RP Hughes GJ The behaviour of peptides on reverse-phase supports during high-pressure liquid chromatography Biochem J 1981 199 31 41 7337711 10.1042/bj1990031 PMC1163331

[B49] Wolfenden R Andersson L Cullis PM Southgate CC Affinities of amino acid side chains for solvent water Biochemistry 1981 20 849 855 10.1021/bi00507a030 7213619

[B50] Zimmerman JM Eliezer N Simha R The characterization of amino acid sequences in proteins by statistical methods J Theor Biol 1968 21 170 201 10.1016/0022-5193(68)90069-6 5700434

[B51] Stirzaker D Elementary probability 2003 2 Cambridge, UK; New York: Cambridge University Press

[B52] Bailey TL Gribskov M Methods and statistics for combining motif match scores J Comput Biol 1998 5 211 221 10.1089/cmb.1998.5.211 9672829

[B53] Bailey TL Gribskov M Combining evidence using p-values: application to sequence homology searches Bioinformatics 1998 14 48 54 10.1093/bioinformatics/14.1.48 9520501

[B54] Hochberg Y A sharper Bonferroni procedure for multiple tests of significance Biometrika 1988 75 800 802 10.1093/biomet/75.4.800

[B55] Simes RJ An improved Bonferroni procedure for multiple tests of significance Biometrika 1986 73 751 754 10.1093/biomet/73.3.751

[B56] Sarkar SK Chang C-K The Simes method for multiple hypothesis testing with positively dependent test statistics Journal of the American Statistical Association 1997 92 1601 1608 10.2307/2965431

[B57] Geisser S Predictive inference : an introduction 1993 New York: Chapman & Hall

[B58] Cattell RB The scree test for the number of factors Multivariate Behavioral Research 1966 1 245 276 10.1207/s15327906mbr0102_10 26828106

[B59] Addington T Calisto B Alfonso-Prieto M Rovira C Fita I Planas A Re-engineering specificity in 1,3-1, 4-beta-glucanase to accept branched xyloglucan substrates Proteins10.1002/prot.22884 21069723

[B60] Gaiser OJ Piotukh K Ponnuswamy MN Planas A Borriss R Heinemann U Structural basis for the substrate specificity of a Bacillus 1,3-1,4-beta-glucanase J Mol Biol 2006 357 1211 1225 10.1016/j.jmb.2006.01.014 16483609

[B61] Olivera-Nappa A Andrews BA Asenjo JA A mixed mechanistic-electrostatic model to explain pH dependence of glycosyl hydrolase enzyme activity Biotechnol Bioeng 2004 86 573 586 10.1002/bit.20063 15129441

