# Supplementary material for: Mutagenesis Objective Search and Selection Tool (MOSST): an algorithm to predict structure-function related mutations in proteins
Source: BMC Bioinformatics. 2011 Apr 27;12:122. doi: 10.1186/1471-2105-12-122 (PMC3123232; doi:10.1186/1471-2105-12-122)
Supplement: Additional file 1 — README FIRST!.pdf (Portable Document Format); file with general information and user instructions. [file 1471-2105-12-122-S1.PDF]

General Information

Files included in the Supplementary Materials

The Supplementary Materials include the following files:

- **README FIRST!.pdf**: this file.
- **MOSST Essential Files.zip**: zipped file including all routines for the MATLAB GUI implementation of MOSST and exemplary alignment files for family 16 glycosyl hydrolases.
- **Basic User Guide.pdf**: user guide with short explanations and instructions to run and operate the MOSST MATLAB GUI.

Software and hardware requirements

In order to run the MATLAB GUI implementation of MOSST, you will need an official MATLAB license including the Statistics Toolbox. The GUI may be run in the OS of your choice, provided that your MATLAB license also runs in the same OS. Your hardware must be compatible with your MATLAB version.

Installation instructions

Follow these instructions to install and run the MATLAB GUI implementation. We assume that you are familiar with MATLAB use. You will need MATLAB version 5 or better in order to run these files.

1) Unzip **MOSST Essential Files.zip** into your preferred folder.
2) The unzipped file contains two folders: **MATLAB files** and **Exemplary alignments.**
3) Put the **MATLAB files** folder into the MATLAB path. It is also recommended that you put the **Exemplary alignments** folder in the MATLAB path, but it is not mandatory.
4) To run the MOSST MATLAB GUI, start your MATLAB software and type MOSST in the MATLAB command window.
5) Follow the Basic User Guide (**Basic User Guide.pdf**) instructions to operate the GUI.

Additional support

If you need further support, please visit the Millennium Institute for Cell Dynamics and Biotechnology (ICDB) website (http://www.icdb.cl) to find more comprehensive user guides and new versions of the GUI implementation, and the latest news about MOSST.

If you have further inquiries or need guidance, please email your questions to the corresponding author (Alvaro Olivera-Nappa, aolivera@ing.uchile.cl) or to the appointed person at http://www.icdb.cl.
