# Supplementary material for: Mutagenesis Objective Search and Selection Tool (MOSST): an algorithm to predict structure-function related mutations in proteins
Source: BMC Bioinformatics. 2011 Apr 27;12:122. doi: 10.1186/1471-2105-12-122 (PMC3123232; doi:10.1186/1471-2105-12-122)
Supplement: Additional file 3 — Basic User Guide.pdf (Portable Document Format); user guide with short explanations and instructions to run and operate the MOSST MATLAB GUI implementation. These additional files and MOSST latest developments can be found at the website of the Millennium Institute for Cell Dynamics and Biotechnology (ICDB) http://www.icdb.cl. [file 1471-2105-12-122-S3.PDF]

Basic User Guide for the MATLAB GUI implementation of MOSST (v1.0, February 2011)

Software and hardware requirements

In order to run the MATLAB GUI implementation of MOSST, you will need an official MATLAB license including the Statistics Toolbox. The GUI may be run in the OS of your choice, provided that your MATLAB license also runs in the same OS. Your hardware must be compatible with your MATLAB version.

Installation instructions

Follow these instructions to install and run the MATLAB GUI implementation. We assume that you are familiar with MATLAB use. You will need MATLAB version 5 or better with the Statistics Toolbox in order to run these files.

1) Unzip **MOSST Essential Files.zip** into your preferred folder.
2) The unzipped file contains two folders: **MATLAB files** and **Exemplary alignments**.
3) Put the **MATLAB files** folder into the MATLAB path. It is also recommended that you put the **Exemplary alignments** folder in the MATLAB path, but it is not mandatory.
4) To run the MOSST MATLAB GUI, start your MATLAB software and type MOSST in the MATLAB command window.

Running the MATLAB GUI implementation of MOSST

The analysis is performed through different windows as follows.

"Selection of the multiple alignment file" window

When running MOSST, a first Windows appears displaying a message to select a multiple alignment file. Type the name of the alignment file in the **Alignment file** cell, including any extension if present. Make sure that the alignment file is in a folder included in the MATLAB path or is in the active MATLAB current directory.

The alignment file must be a text only file with the format of the exemplary alignment files included in **MOSST Essential Files.zip**. Each line in the file has the following structure:

(**Protein name**)**Alignment**

where **Protein name** is a name describing the corresponding protein and **Alignment** is an amino acid sequence in one-letter code including gaps that are represented by hyphens (-), with no spaces or other characters. The format of this alignment is equivalent to the format of the alignment provided by ClustalW and other similar multiple alignment programs. The length of **Protein name** must not be equal for

every line in the file, but the length of the **Alignment** must be exactly the same for all lines in the alignment file. This length is the maximum length of the alignment.
The "Selection of the multiple alignment file" window also includes a second cell to input the number of the target protein in the multiple alignment for which mutation probabilities will be calculated. Press **Continue** to proceed to the next window.
Example: for all the example files included, the target protein is the first sequence in the alignment files, so this parameter must be set to 1.

"Redundancy removal in the multiple alignment" window

The next Windows shows a dendrogram plot with the classification of the proteins in the alignment according to their similarity and an agglomeration distance plot that shows the contiguous distances between the proteins in the alignment, ordered from lowest to highest similarity (left to right). Proteins are identified by their sorting number in the alignment file.
If you wish to change the alignment file, press **Change** in the Change alignment box.
The plots are used to set a threshold to remove redundancy in the protein alignment and keep only representative proteins for further analysis. The threshold is plotted as a red line. All proteins with a similarity lower than the threshold similarity value are conserved, whereas the remaining are discarded.
The default threshold value is 0.85 (discard proteins that share more than 85% similarity). This value can be changed by entering the selected value in the **Critical similarity** cell and pressing the **Plot** button to see the results. To restore the plots to the original values, press the **Restore** button.
Use the plots to orient your selection to eliminate redundancy in the protein set. Ideally, you should look for the threshold line to traverse the highest leftmost step in the agglomeration plot. Once you are satisfied with your threshold value, press **Continue** to proceed to the next window.
The plots can be printed or exported as image files by using the buttons in the **Print or export** box.
Example: for the "Alignment.txt" exemplary file, a threshold value of 0.85 is considered good to remove redundant proteins from the alignment.

"Plot of the differences between successive values" window

The following window shows a plot of the differences between significance values calculated for each position of the alignment. To calculate these differences, the value of the difference between the lowest significance value for some position in the alignment with the second lowest significance value is calculated, then the difference between the second and the third lowest significance values and so on. The differences are plotted in this order from left to right and moving average smoothing lines are calculated with different window sizes, ranging from 1 (no smoothing) to 7.
The plot can be zoomed between any two positions in the abscissa axis by introducing the limits in the cells below the plot and pressing **Plot**.
The plot can be printed or exported as an image file by using the buttons in the **Print or export** box.
A critical Negative Logarithmic Significance Difference Value (NLSDV) must be selected to proceed to the next step of the analysis. This value indicates the

significance logarithmic threshold under which a significance value in a given position is marked as highly significant and therefore that position must be labelled as a primary position.
To select a good NLSDV, the highest intermediate-rightmost peak has to be identified in the plot and the position immediately to its left (read from the abscissa axis) must be introduced in the cell inside the **Selected critical position** box. The specific critical NLSDV is displayed when pressing the **Critical NLSDV** button. The selected value is a matter of choice and is not mandatorily the value associated to the highest peak, but we encourage you to stick to this premise to achieve statistically consistent results. A more
Once you are satisfied with your critical NLSDV value, press **Continue** to proceed to the next window.
Example: for the "Alignment.txt" exemplary file, the optimal critical NLSDV is 3.7904 (obtained by selecting position 330 of the plot).

"Plots for the determination of mutation targets" window

This window shows two plots that indicate the final result of the classification of positions in the multiple alignment according to their significance calculated by MOSST. Note the logarithmic scale in the abscissa axis. The top plot shows every alignment position classified as non-significant, significant, very significant and primary positions, according to the definitions given in the paper, as a function of the total significance for each position, plotted as differences in a log-scale (NLSDV) in the ordinate axis. The bottom plot shows the significances for each component (property vector) at each position, also in log-scale (NLSV) for the ordinate axis.
The plots can be zoomed as previously explained by changing the starting and final positions. The alignment can also be changed once again by pressing the **Change** button in the **Change alignment** box. The plots can also be printed or exported as image files by using the buttons in the **Print or export** box.
The critical NLSDV can be manually entered in the **Change critical NLSDV** box cell if desired. The original value calculated in the previous window can be restored by pressing the **Restore** button.
Mutation probabilities can be calculated for any position in the alignment by introducing the position number in the **Mutation possibilities** box and pressing the **OK** button. This can be done for every type of position (i.e. is not restricted only to primary or very significant positions). This pops up a new window.
To finish the analysis, press the **End** button.

"Determination of the mutation possibilities" window

When selecting a position number in the **Mutation possibilities** box of the previous window, this new window is displayed showing a bar plot (similar in function to a scree plot) with the probability of occurrence of each amino acid calculated for the selected position and sorted from highest (top) to lowest (bottom). On top of this plot, a left box indicates the number of the selected position in the multiple alignment and a right box shows the amino acid that occupies this position in the target protein (selected in the first window).

At the right side of the plot, a table shows the calculated probabilities of occurrence of each amino acid in the selected position, tabulated and sorted from highest (top) to lowest (bottom). The most significantly frequent amino acids in the selected position are highlighted in red. Improbable amino acids are listed as having zero probability of occurrence.

This data can be printed or exported as image files by pressing the **Export** button.

When done, the user can return to the previous window by pressing the **OK** button.

Additional support

If you need further support, please visit the Millennium Institute for Cell Dynamics and Biotechnology (ICDB) website (http://www.icdb.cl) to find more comprehensive user guides and new versions of the GUI implementation, and the latest news about MOSST.

If you have further inquiries or need guidance, please email your questions to the corresponding author (Alvaro Olivera-Nappa, aolivera@ing.uchile.cl) or to the appointed person at http://www.icdb.cl.
